# Mortality and years of life lost due to pancreatic cancer in China, its provinces, urban and rural areas from 2005 to 2020: results from the national mortality surveillance system

**DOI:** 10.1186/s12885-023-11258-7

**Published:** 2023-09-21

**Authors:** Yangyang Xu, Wei Liu, Zheng Long, Lijun Wang, Maigeng Zhou, Peng Yin

**Affiliations:** National Center for Chronic and Noncommunicable Disease Control and Prevention, Chinese Center for Disease Control and Prevention, 27 Nanwei Road, Xicheng District, Beijing, 10050 China

**Keywords:** Pancreatic cancer, Mortality, Years of life lost, Decomposition analysis, Disease burden, China, Urban and rural areas, Provinces

## Abstract

**Background:**

Pancreatic cancer is a growing public health concern in China, and depicting it from different perspectives would provide a comprehensive understanding of its epidemiological characteristics.

**Methods:**

Data from the National Mortality Surveillance System (NMSS) in China was used to estimate the number of deaths, years of life lost (YLL), age-standardized mortality rate (ASMR) and age-standardized YLL rate in China, its provinces and urban-rural areas from 2005 to 2020. Joinpoint regression analysis was employed to explore the temporal trends of ASMR and age-standardized YLL rate. Decomposition analysis was conducted to assess the contribution of population growth, population aging and cause-specific mortality rate to the increment of pancreatic cancer deaths.

**Results:**

A total of 100,427 pancreatic cancer deaths and 2,166,355 pancreatic cancer related YLL were estimated in China in 2020. The overall ASMR significantly increased from 6.6/100 000 in 2005 to 7.4/100 000 in 2020, and was higher in men than that in women. Age-standardized YLL rate showed a similar trend. The mortality rates of pancreatic cancer were generally higher in northeast China than in southwest China. The highest ASMRs were found in Jilin, Zhejiang, Inner Mongolia and Anhui, and the lowest ones in Guangxi, Yunnan, Tibet, and Hainan. The disease burden due to pancreatic cancer presented a significant upward trend in rural areas and a downward trend in urban areas.

**Conclusions:**

The burden associated with pancreatic cancer had been increasing in China from 2005 to 2020. The escalating disease burden of pancreatic cancer in rural areas necessitates the implementation of effective control and prevention measures. Relevant provinces should pay greater attention to the prevailing of pancreatic cancer, particularly those exhibiting higher mortality rates.

**Supplementary Information:**

The online version contains supplementary material available at 10.1186/s12885-023-11258-7.

## Background

Pancreatic cancer is known as one of the most fatal cancers for its malignancy, aggressiveness and low survival rate, and its mortality is almost identical to incidence [1]. According to GLOBOCAN2020, pancreatic cancer was the 12th most common malignancy and 7th leading cause of cancer mortality [2]. The highest mortality rates of pancreatic cancer are usually observed in developed countries, ranking as the 4th leading cause of death among all cancers in the United States [3] and 6th in Europe [4]. Although the rank is usually outside top 10 in most developing countries, the incidences are gradually catching up with that in developed countries with the westernization of lifestyles in some developing countries [5], including China. In 2017, the number of new cases and deaths of pancreatic cancer in China were 83.6 thousand and 85.1 thousand, accounting for one-fifth of all pancreatic cancer new cases and deaths globally and increased by 230.94% and 236.08% compared with 1990, respectively [6]. With China’s modernization process, the burden caused by pancreatic cancer might increase to a higher level over the next few decades.

In China, pancreatic cancer has not received as much public attention as other types of cancer. Previous studies on the burden of pancreatic cancer in China produced similar results, with an increasing trend on both incidence and mortality and higher rates in men than in women [7, 8]. However, few of these studies focused on the burden of pancreatic cancer and its trend at the provincial level, and the evidence on the differences between urban and rural areas and how the differences changed over time. Therefore, it is important to update the overall situation of the disease burden caused by pancreatic cancer to get more attention from policy makers and researchers in China and other developing countries.

Based on the data from the NMSS, we conducted this study to examine the temporal trends of pancreatic cancer mortality and years of life lost (YLL) in China, its provinces, urban and rural areas during 2005 to 2020, to explore the differences of disease burden due to pancreatic cancer between urban and rural areas and among mainland provinces and the causes of the increment of pancreatic cancer deaths during the study period.

## Methods

### Data source

The data of pancreatic cancer in our study was derived from the NMSS. which covered 24.3% of the total population of China from 605 disease surveillance points (DSPs) and was well representative at the national and provincial level. Detailed descriptions of NMSS were published elsewhere [9]. Briefly, the NMSS collected deaths occurred in hospitals and outside of hospitals across all mainland provinces in China. Data from the NMSS has been widely used for burden of disease estimation and policy formulation. Under-reporting data which was obtained from the retrospective under-reporting field surveys for the NMSS conducted in 2009, 2012, 2015 and 2018, was used to adjust the mortality rate of pancreatic cancer [10]. In consideration of the relatively high under-reporting rate of children in the NMSS, we used the Under-5 Mortality Rate(U5MR) at the country level from the Global Burden of Disease Study as a separate data source [11]. The urban or rural area was defined by the residential address of the deceased person. Urban areas included districts in municipalities or prefecture-level cities, and rural areas included counties and county-level cities. Population information at the surveillance points was obtained from China’s National Bureau of Statistics. In addition, we used demographic data from 2020 census to standardize the population structure for each calendar year during the study period.

### Mortality rate estimation

In the NMSS, a small proportion of the codes for cause of death are garbage codes defined as deaths with non-specific codes, deaths that could not be underlying causes of death, or deaths assigned to intermediate but not underlying causes of death. We redistributed those garbage codes to the most likely cause of death by age, sex, location and year using method developed by Naghavi and his colleagues [12]. Then, we calculated the proportion of pancreatic cancer (ICD-10 codes: C25.0-C25.9) in the NMSS by provinces-age-gender. The mortality rate of pancreatic cancer was calculated for each year, location, gender, and age group by multiplying the all-cause mortality rate by the proportion of deaths due to pancreatic cancer from 2005 to 2018.Finally, assuming a steady change in the mortality rate from 2005 to 2018, we fitted a generalized linear model to predict the mortality rate by location, sex, and age group in 2019 and 2020.

#### YLL Estimation (per 100,000 population)

Years of life lost (YLL) is a measure of premature mortality that takes into account both the frequency of deaths and the age at which it occurs, giving greater weight to deaths at younger age and lower weight to deaths at older age, and are calculated from the number of deaths multiplied by a standard life expectancy at the age at which death occurs. In our study, we used a theoretical minimal risk reference life table to calculate YLL of pancreatic cancer during 2005 to 2020 [12].

### Statistical analysis

Age-standardized rate (per 100,000) of pancreatic cancer was calculated by the direct age standardization using the 7th China census in 2020 as the standard population. Joinpoint regression analysis was performed to calculate the average annual percent change (AAPC) and corresponding 95% confidence intervals (CIs) of age-standardized mortality rate and age-standardized YLL rate, which assessed the direction and magnitude of general trend over time. Using methods adapted from demographic research from Das Gupta [13], we decomposed variation in numbers of deaths of PC from 2005 to 2020, using three explanatory components: change in the growth of the total population; shifts in the population structure by age and changes in the pancreatic cancer mortality rates. All analyses were carried out with SAS (Version 9.4, SAS Institute Inc., Cary, NC, USA) and Joinpoint Regression Program (Version 4.9.0.1, National Cancer Institute, Rockville, MD, US). Graphic drawing was plotted with R (Version 4.3.1, The R Foundation for Statistical Computing, Vienna, Austria).

## Results

In 2020, a total of 100,427 pancreatic cancer deaths (63,104 men, 37, 323 women) and 2,166,355 pancreatic cancer related YLL (1,391,322 men, 775,023 women) were estimated in China. The ASMR increased from 6.6/100 000 in 2005 to 7.4/100 000 in 2020, and age-standardized YLL rate increased from 146.2/100 000 in 2005 to 158.6/100 000 in 2020 (Table 1). The ASMR and age-standardized YLL rate were consistently significantly higher in men than those in women.

**Table 1:** Estimated Deaths and YLL Due to Pancreatin Cancer in China

	Number of Death	Age-Standardized Mortality Rate (1/100,000)	Number of YLL	Age-Standardized YLL Rate (1/100,000)
**Men**				
2005	33,550	8.7	800,188	192.2
2010	40,840	9.2	943,436	200.7
2015	48,687	9.7	1,092,988	207.5
2020	63,104	10.0	1,391,332	212.4
AAPC(%)		0.95 (0.89 to 1.00)*		0.66 (0.60 to 0.72)^*^
**Women**				
2005	20,124	4.7	451,300	102.3
2010	24,354	4.9	531,180	106.0
2015	28,653	5.1	611,360	108.3
2020	37,323	5.2	775,023	108.7
AAPC(%)		0.64 (0.49 to 0.78)^*^		0.42 (0.36 to 0.47)^*^
**Total**				
2005	53,674	6.6	1,251,488	146.2
2010	65,194	7.0	1,474,616	152.3
2015	77,340	7.3	1,704,348	157.0
2020	100,427	7.4	2,166,355	158.6
AAPC(%)		0.76 (0.58 to 0.95)^*^		0.54 (0.47 to 0.62)^*^

Note: ^*^p < 0.001

Abbreviations: YLL, years of life lost; AAPC, average annual percent change;

**Fig. 1:**
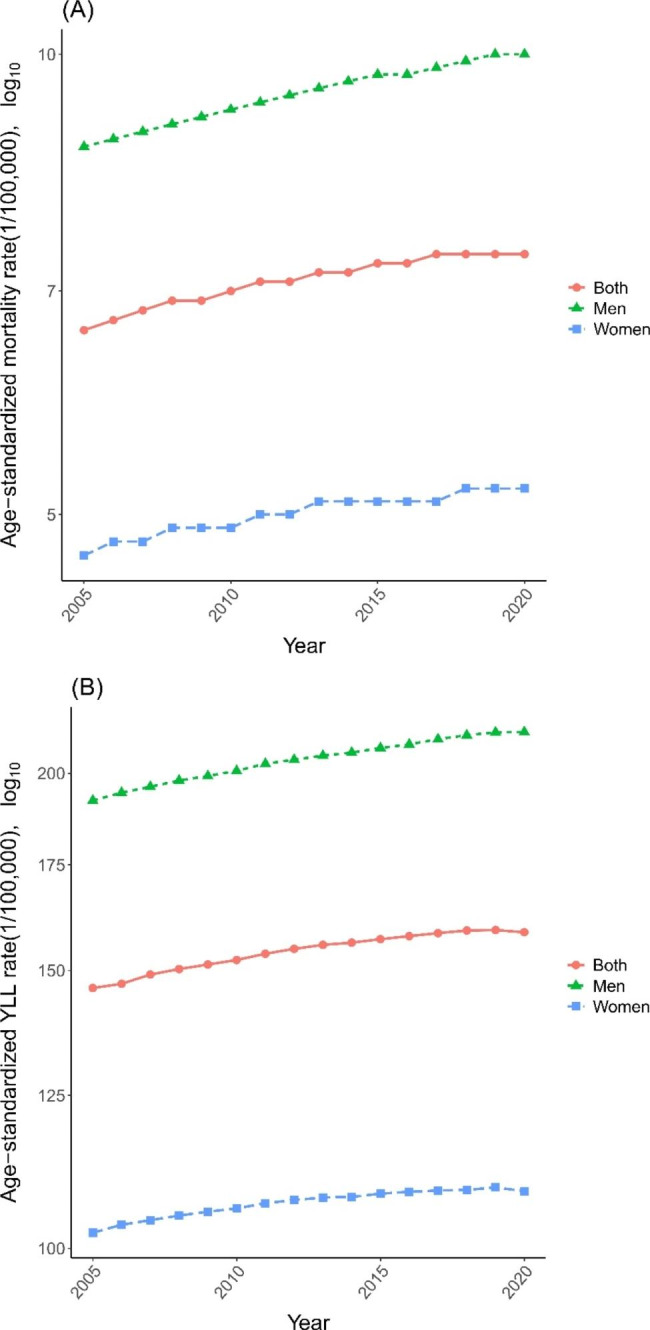
**(A)** Age-standardized mortality rate of pancreatic cancer in China from 2005 to 2020 **(B)** Age-standardized YLL rate of pancreatic cancer in China from 2005 to2020

The ASMR of pancreatic cancer increased significantly in both men (AAPC = 0.95, 95%CI: 0.89 to 1.00) and women (AAPC = 0.64, 95%CI:0.49 to 0.78) during 2005 to 2020 in China, with an increment of 14.9% and 10.6%, respectively (Fig. 1A; Table 1). The age-standardized YLL rate shows a similar increasing trend (Fig. 1B and Table 1).

**Fig. 2:**
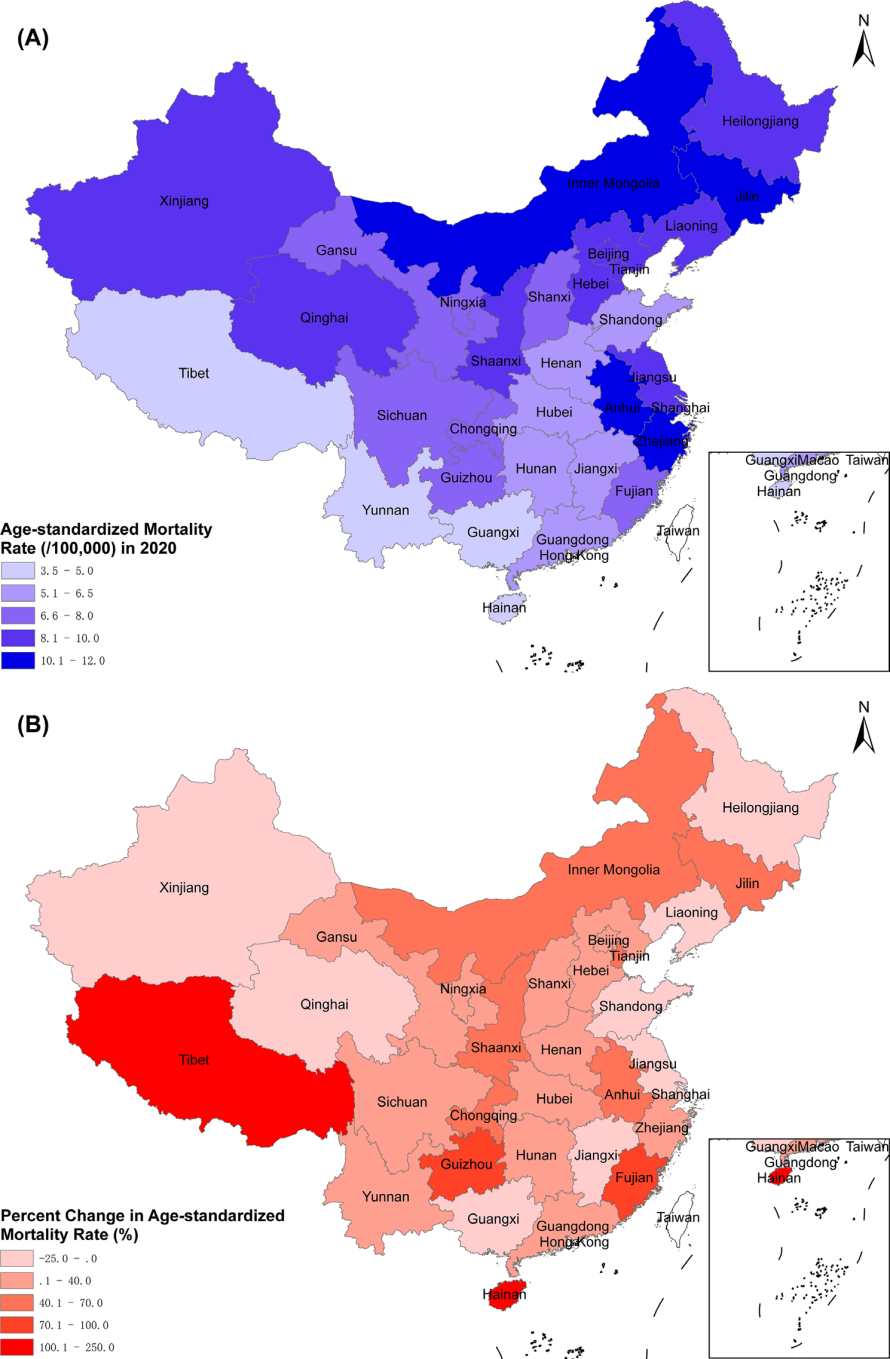
**(A)** Age-standardized mortality rate of pancreatic cancer by province in 2020. **(B)** Percent change in age-standardized mortality rate of pancreatic cancer by province from 2005 to 2020

As shown in Fig. 2A and Table S1, the highest ASMR were in Jilin (11.9/100,000), Zhejiang (11.6/100,000), Inner Mongolia (11.0/100,000), and Anhui (10.6 /100,000) in 2020. Guangxi (3.5/100,000), Yunnan (4.4/100,000), Tibet (4.9/100,000) and Hainan (4.9/100,000) had the lowest ASMRs. During 2005 to 2020, Tibet (+ 250.0%), Hainan (+ 104.2%) and Guizhou (+ 78.4%) showed the largest increase of ASMR. The largest decline of ASMR was observed in Xinjiang (25.0%), Shandong (24.7%), and Shanghai (24.2%) (Fig. 2B and Table S2).

**Fig. 3:**
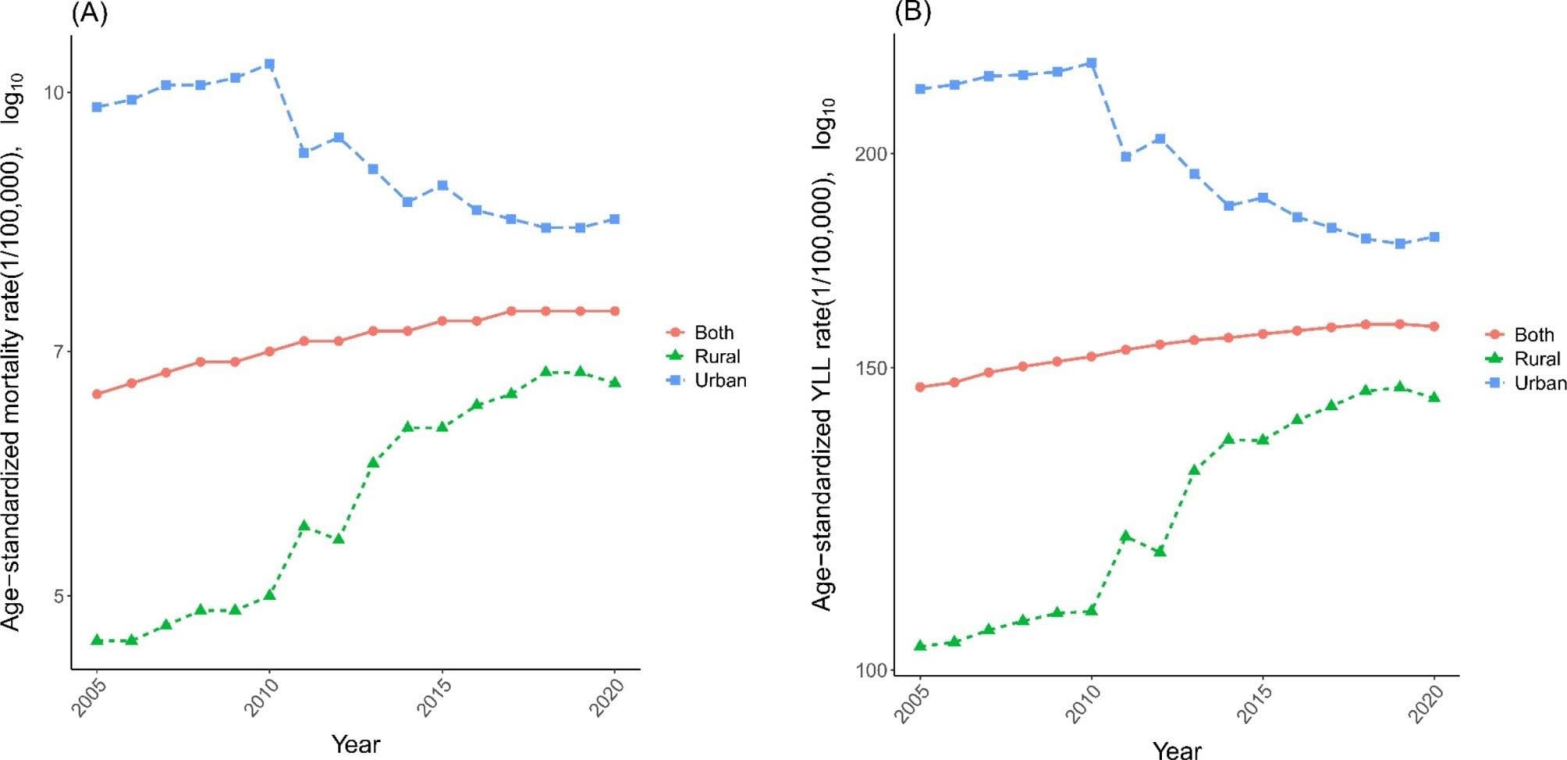
**(A)** ASMR trend of pancreatic cancer in urban and rural areas from 2005 to 2020 **(B)** Trends of the age-standardized YLL rate of pancreatic cancer in urban and rural areas

Nationally, the ASMR presented a downward trend (AAPC = 1.11, 95%CI: 2.12 to 0.10) from 9.8/100,000 in 2005 to 8.4/100,000 in 2020 in urban areas and an upward trend (AAPC = 2.59, 95%CI: 1.54 to 3.64) from 4.7/100 000 in 2005 to 6.7/100 000 in 2020 in rural areas (Fig. 3A and Table S3). Age-standardized YLL rate showed a similar trend in urban and rural areas (Fig. 3B and Table S4). In 2020, the highest ASMR in urban areas were in Xinjiang (15.3/100,000), Zhejiang (13.8/100,000) and Qinghai (13.3/100,000). Jilin (12.0/100,000), Zhejiang (11.3/100,000), Inner Mongolia (11.2/100,000) and Anhui (11.1/100,000) showed the highest ASMRs in rural areas. Different secular patterns and disparity of ASMR were observed in urban and rural areas across the 31 mainland provinces. Yunnan, Xinjiang and other 16 provinces showed a downward trend of the ASMR in urban areas. Guizhou, Inner Mongolia and other 22 provinces showed an upward trend of the ASMR in rural areas (Table S3).

**Fig. 4:**
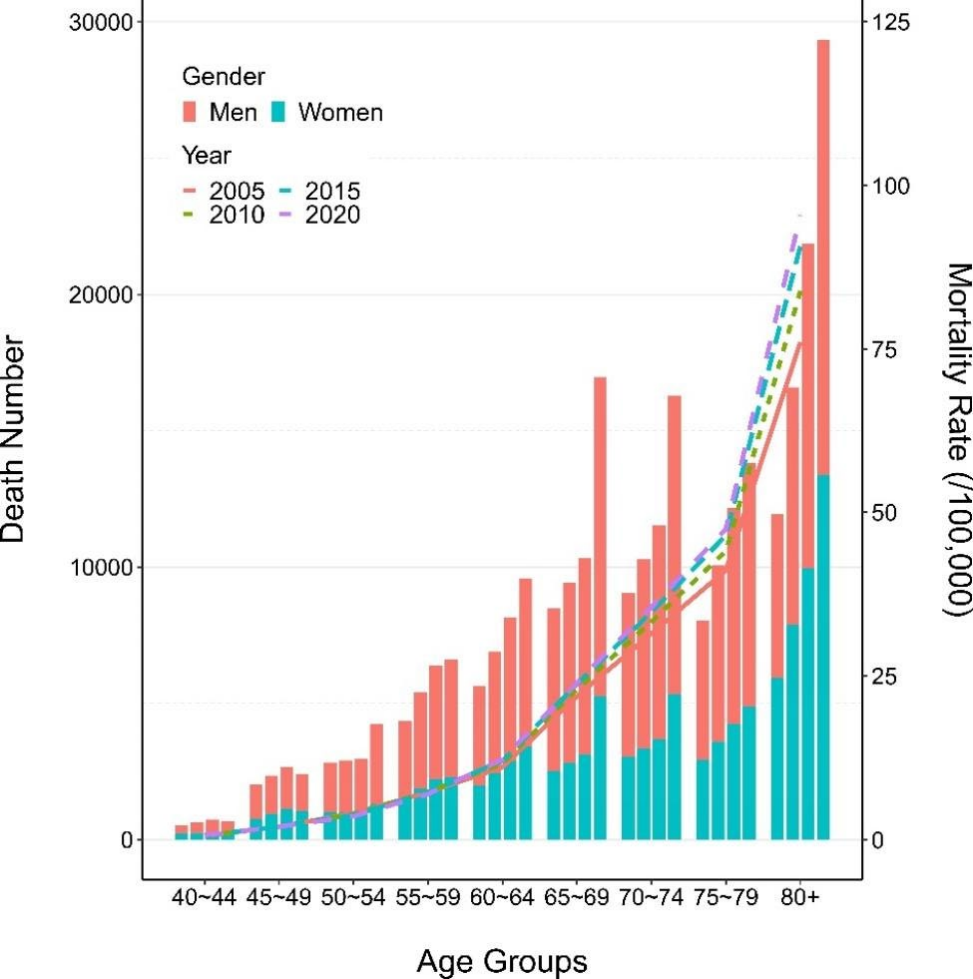
Death numbers of each age group in 2005, 2010, 2015, and 2020 (bars). Mortality rate of each age group in 2005, 2010, 2015, and 2020 (lines)

Figure 4 shows the trends of pancreatic cancer mortality rate and death numbers in different age groups in 2005, 2010, 2015 and 2020. It is obvious that the mortality rate increased with age, with two marked inflection points at the age groups of 60 to 64 and 75 to 79 in each year. The ASMR increased slowly in the age group of less than 60, and increased sharply in the age group greater than 60. The death numbers increased with age and peaked at the agegroup of over 80 in both men and women.

**Fig. 5:**
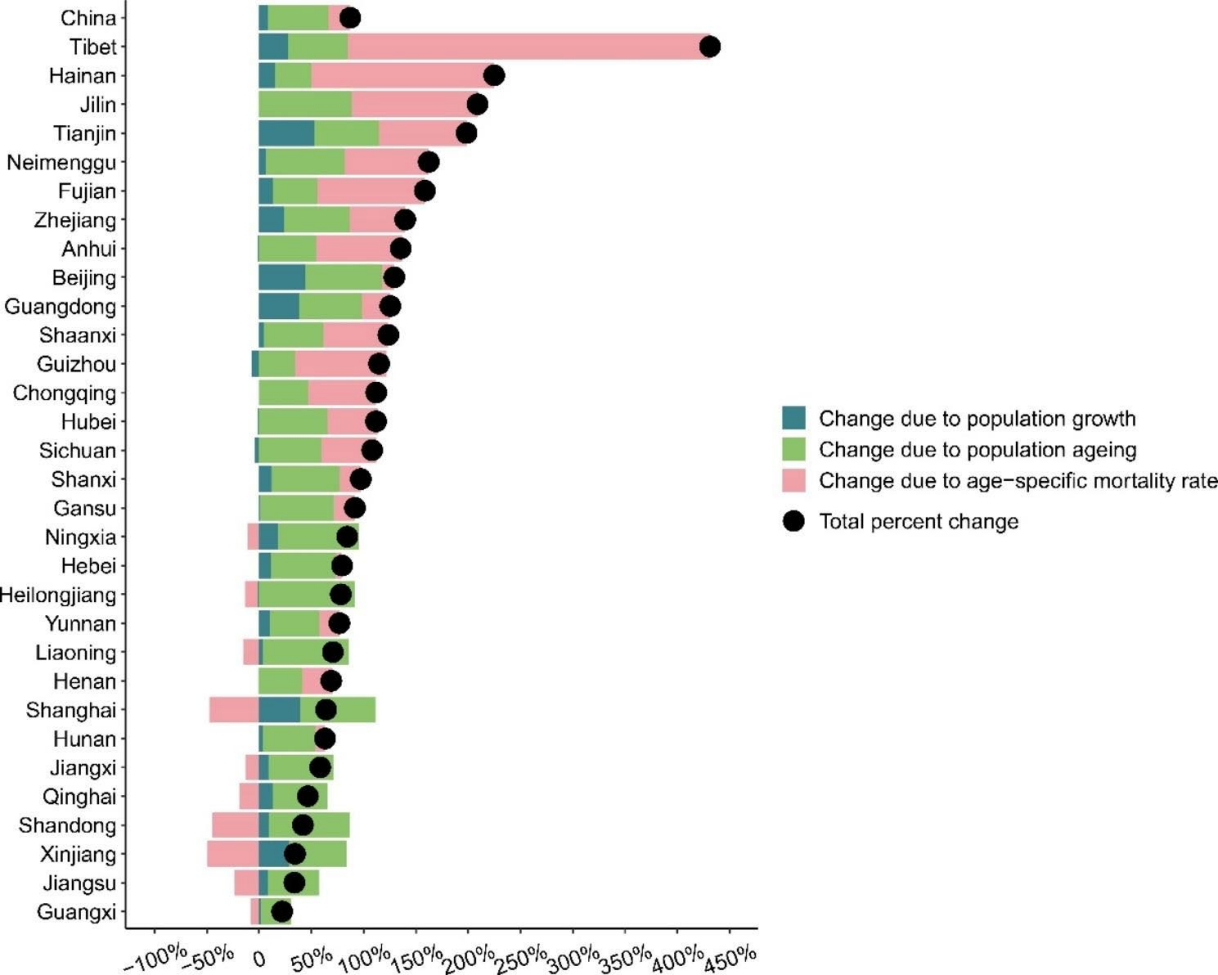
Increment in pancreatic cancer deaths due to the changes in population growth, population aging, and age-specific mortality rate in China and its 31 provinces from 2005 to 2020

The decomposition analysis of increment of death numbers between 2005 and 2020 are showed in Fig. 5. At the national level, the three aspects all increased significantly. Compared with 2005, the total number of deaths due to pancreatic cancer in 2020 increased by 87.1%, of which the increase in mortality rate accounted for 20.7%, population growth accounted for 8.5%, and population aging accounted for 57.9%. At the provincial level, all provinces had an increase in death numbers. The provinces with the greatest contribution to the increase in pancreatic cancer mortality rate were Tibet (+ 346.9%), Hainan (+ 175.2%), Jilin (+ 120.7%), and those with the lowest contribution were Xinjiang (49.3%), Shanghai (47.3%) and Shandong (44.9%). The provinces with the greatest contribution to the increase in population growth were Tianjin (+ 52.5%), Beijing (+ 44.2%), Shanghai (+ 39.4%), and the provinces with the lowest contribution were Guizhou (7.2%), Sichuan (4.0%) and Heilongjiang (1.7%). The provinces with the greatest contribution to the increase in population aging were Heilongjiang (+ 91.5%), Jilin (+ 88.8%), Liaoning (+ 82.6%) and those with the lowest contribution were Guangxi (+ 28.7%), Hainan (+ 34.1%) and Guizhou (+ 34.6%).

## Discussion

We observed a significant increase in the ASMR and age-standardized YLL rate due to pancreatic cancer in China during 2005 to 2020. There were substantial variations in ASMRs among provinces in 2020, ranging from 3.5/100,000 in Guangxi to 11.6/100,000 in Jilin. The magnitude of variations varied markedly from − 25.0% in Xinjiang to + 250.0% in Tibet. The age-standardized YLL rates varied among provinces, ranging from 78.9/100,000 in Guangxi to 248.6/100,000 in Zhejiang. The gaps of ASMRs and age-standardized YLL rates between urban and rural areas gradually narrowed over time.

Consistent with the previous studies [7, 8], the ASMR increased with age and year, and was higher in men than that in women. The higher ASMR in men might be attributed to men’s higher level of occupational exposure and unhealthy habits such as smoking and alcohol drinking in China. According to GLOBOCAN2020, the mortality of pancreatic cancer was higher in Europe (8.2/100,000 in Germany, 7.7/100,000 in France, 5.9/100,000 in the UK) and North America (6.6/100,000 in the US, 6.1/100,000 in Canada) [14], and the ASMR in China was 5.1/100,000, ranking outside top 50. YLL is a metric that reflect the burden caused by pancreatic cancer and is widely used for public surveillance. It offers opportunity to quantify the level and trend of premature mortality and to identify the leading cause of premature deaths. The results from the GBD database showed that the age-standardized YLL rates due to pancreatic cancer in China had exceeded the average level around the world since 2004. In addition to the increasing incidence and mortality rate, poor diagnostic capacity and lack of effective treatment might result in international disparity. The increasing trend for death due to pancreatic cancer in China may be largely attributed to the population aging and behavioral changes during recent decades. In addition, improvements in diagnose and cancer registration practices may also be in play [15].

Over the past decades, China has undergone rapid social and economic development, accompanied by a significant increase in the prevalence of modifiable risk factors associated with pancreatic cancer. Smoking is a well-established risk factor for pancreatic cancer [16–18]. WHO estimated that there were about 315 million smokers in 2015 in China, around 28% of the adult population [19]. Moreover, the age of smoking initiation has shifted forward substantially over time. A pooled study based on China Health and Nutrition Survey found that the average age of smoking initiation decreased significantly from 22.0 to 17.5 during recent decades [20]. Obesity or increased weight showed a positive association with pancreatic cancer. Obesity prevalence has been steadily increasing since1980s in China. Wang and his colleagues found that standardized mean body mass index (BMI) levels rose from 22·7 kg/m² in 2004 to 24·4 kg/m² in 2018 and obesity prevalence increased from 3·1% to 8·1% [21]. Ma et al. demonstrated that the overall distribution curve of BMI significantly moved to the right between 1993 and 2015 [22]. The association between pancreatic cancer and alcohol consumption varied from different studies [23–25]. However, heavy alcohol consumption is associated with pancreatitis, and pancreatitis is a convincing risk factor for pancreatic cancer [26].

Compared to other types of cancer, pancreatic cancer is a rare tumor in China. However, there are certain areas with relatively higher mortality rates. Our study found that ASMRs and age-standardized YLL rates were higher in the northeast China than those in the southwest China, which was similar to the distribution of the prevalence of obesity and diabetes [27, 28]. The variation in smoking prevalence among provinces offers a limited explanation for the disparities observed in ASMRs and age-standardized YLL rates between them. For example, Yunnan province, which has the highest smoking rate (35.7%) in China, has a lower ASMR (4.4 per 100,000) and age-standardized YLL rate (103.6 per 100,000); Shanghai, which has the lowest smoking rate (17.8%), has a higher ASMR (9.1 per 100,000) and age-standardized YLL rate (163.5 per 100,000). The potential cause for this phenomenon could be attributed to the inherent bias (ecological fallacy) in interpreting provincial mortality rates based on risk factors at the provincial level. Other risk factors, such as high-fat/high-protein diets and psychological distress, may also contribute to the increased mortality rates of pancreatic cancer. Chan et al. found that high-protein/high-fat diet could increase the risk of catching pancreatic cancer [29]. In pastoral areas, such as Inner Mongolia, Xinjiang and Qinghai, herders consume more beef and mutton compared to other regions. This dietary pattern might be one reason for the high mortality rates. Batty et al. pooled 16 prospective cohort studies and found that psychological distress increased the risk of death from pancreatic cancer [30]. This suggests that psychological distress may contribute to the higher mortality rates from pancreatic cancer observed in economically developed provinces such as Beijing, Shanghai and Zhejiang. In addition, the degree of population aging tends to be higher in provinces with high ASMRs and age-standardized YLL rates [31].

The urban-rural disparity varied among provinces. The main reasons for the trends of ASMRs and age-standardized YLL rates in urban areas might be due to the fact that residents are more aware of their health conditions and have better access to medical services, thereby reducing the risk of developing pancreatic cancer. The observed trend for rural areas might be associated with the wider coverage of basic medical insurance in China and the residents in rural areas can afford to seek medical support. Further, there is a higher degree of population aging observed in rural areas compared to urban areas due to young individuals migrating from rural areas to urban areas [31].

Our study revealed that population aging was the main driver for the increment of death numbers between 2005 and 2020, accounting for 66.5% of the total increase, although the increase varied among provinces. For provinces with a higher proportion of older people, such as Heilongjiang, Jilin, Liaoning, Shanghai and Shandong, population aging played a significant role in the increase in death numbers. For Tibet and Hainan, age-specific mortality was the major driver for the increase in death numbers. This might be due to the underreporting of death data in the early days and improvement in the later days. For Shanghai, Tianjin and Beijing, population growth also acted as an important role, because they were all among the cities which had the largest net inflow population during the past decades.

Population aging is an inevitable challenge in China, and effectively controlling the pancreatic cancer associated risk factors remains a formidable task. Unless there are breakthroughs in diagnostic and therapeutic techniques, the disease burden associated pancreatic cancer is likely to continue increasing for some time. The screening for pancreatic cancer maybe a viable way to cover the shortage in early diagnosis and treatment. However, due to the low incidence of pancreatic cancer and the difficulty in generalization of screening tests in general population, screening for pancreatic cancer is not recommended [32]. Therefore, raising public awareness of pancreatic cancer and increasing the investment on research are imperative at present.

Our study has some limitations. Firstly, underreporting in the National Mortality Surveillance System has always been a problem. Usually, the problem is not caused by reporters, but by the patients who do not go to hospital even after they have related symptoms, and mainly occurs in less-developed areas in China. However, we used under-reporting rate derived from periodical under-reporting field surveys to adjust the mortality rate and the adjusted rate provided more reliable evidence. Secondly, there were some changes in the quality of data during the study period, such as the coding practice in some rural areas. Finally, due to lack of data of lifestyle and metabolic risk factors at the provincial level, we were not able to comprehensively examine the reasons for the differences in the ASMR among different provinces.

## Conclusion

In summary, there were significant increases in the mortality and YLL rate of pancreatic cancer in China between 2005 and 2020. The significant urban-rural disparity suggested that effective control and prevention measures should be taken in the rural areas to reduce the burden caused by pancreatic cancer.

### Electronic supplementary material

Below is the link to the electronic supplementary material.

Supplementary Material 1

## Data Availability

NMSS data and research materials will be made available to other researchers from the corresponding author upon reasonable request.

## Abbreviations

YLL: Years of life lost
NMSS: National Mortality Surveillance System
ASMR: Age-standardized mortality rate
AAPC: Average annual percent change
GLOBOCAN2020: Global Cancer Statistics 2020
U5MR: Under-5 Mortality Rate
ICD: International Classification of Diseases
