# Supplementary material for: Mortality and years of life lost due to pancreatic cancer in China, its provinces, urban and rural areas from 2005 to 2020: results from the national mortality surveillance system
Source: BMC Cancer. 2023 Sep 21;23:893. doi: 10.1186/s12885-023-11258-7 (PMC10512506; doi:10.1186/s12885-023-11258-7)
Supplement: Supplementary file 1 — Supplementary Material 1 [file 12885_2023_11258_MOESM1_ESM.docx]

| **Table S1** The age-standardized mortality rates (1/100,000) by province, gender, 2005-2020 | | | | | | | |
| --- | --- | --- | --- | --- | --- | --- | --- |
| **Provinces** | **Gender** | **2005** | **2010** | **2015** | **2020** | **AAPC** | **Percent Change** |
| China | Both | 6.6 | 7.0 | 7.3 | 7.4 | 0.76(0.58, 0.95) | 12.1% |
| China | Women | 4.7 | 4.9 | 5.1 | 5.2 | 0.64(0.49, 0.78) | 10.6% |
| China | Men | 8.7 | 9.2 | 9.7 | 10.0 | 0.95(0.89, 1.00) | 14.9% |
| Beijing | Both | 7.7 | 7.9 | 8.0 | 8.1 | 0.37(0.29, 0.46) | 5.2% |
| Beijing | Women | 5.7 | 5.8 | 5.9 | 6.0 | 0.37(0.30, 0.44) | 5.3% |
| Beijing | Men | 10.2 | 10.5 | 10.5 | 10.6 | 0.27(0.05, 0.50) | 3.9% |
| Tianjin | Both | 6.6 | 7.5 | 8.4 | 9.4 | 2.39(2.26, 2.53) | 42.4% |
| Tianjin | Women | 6.0 | 6.5 | 7.0 | 7.3 | 1.33(1.26, 1.40) | 21.7% |
| Tianjin | Men | 7.3 | 8.7 | 10.1 | 11.5 | 3.09(2.88, 3.30) | 57.5% |
| Hebei | Both | 7.7 | 8.0 | 8.2 | 8.1 | 0.35(0.25, 0.44) | 5.2% |
| Hebei | Women | 4.5 | 4.4 | 4.3 | 4.2 | -0.52(-0.61, -0.43) | -6.7% |
| Hebei | Men | 11.3 | 12.0 | 12.5 | 12.7 | 0.77(0.67, 0.88) | 12.4% |
| Shanxi | Both | 6.5 | 7.0 | 7.2 | 7.3 | 0.78(0.63, 0.93) | 12.3% |
| Shanxi | Women | 4.0 | 4.3 | 4.6 | 4.8 | 1.12(0.93, 1.30) | 20.0% |
| Shanxi | Men | 9.2 | 9.7 | 9.9 | 10.0 | 0.54(0.48, 0.60) | 8.7% |
| Inner Mongolia | Both | 7.4 | 8.8 | 10.0 | 11.0 | 2.70(2.53, 2.87) | 48.6% |
| Inner Mongolia | Women | 5.0 | 5.9 | 6.7 | 7.3 | 2.58(2.34, 2.83) | 46.0% |
| Inner Mongolia | Men | 9.8 | 11.7 | 13.5 | 14.9 | 2.85(2.71, 3.00) | 52.0% |
| Liaoning | Both | 9.6 | 9.4 | 9.2 | 8.8 | -0.57(-0.66, -0.49) | -8.3% |
| Liaoning | Women | 6.9 | 6.8 | 6.7 | 6.4 | -0.48(-0.60, -0.35) | -7.2% |
| Liaoning | Men | 12.5 | 12.1 | 11.9 | 11.5 | -0.54(-0.50, -0.59) | -8.0% |
| Jilin | Both | 7.2 | 8.9 | 10.5 | 11.9 | 3.54(3.29, 3.62) | 65.3% |
| Jilin | Women | 4.4 | 5.5 | 6.4 | 7.3 | 3.46(3.21, 3.70) | 65.9% |
| Jilin | Men | 10.0 | 12.6 | 14.9 | 17.2 | 3.70(3.56, 3.84) | 72.0% |
| Heilongjiang | Both | 9.3 | 9.1 | 9.1 | 8.9 | -0.33(-0.55, -0.11) | -4.3% |
| Heilongjiang | Women | 5.9 | 6.0 | 6.1 | 6.1 | 0.26(0.16, 0.36) | 3.4% |
| Heilongjiang | Men | 12.8 | 12.4 | 12.3 | 12.0 | -0.38(-0.42, -0.33) | -6.3% |
| Shanghai | Both | 12.0 | 11.4 | 10.3 | 9.1 | -1.83(-1.95, -1.71) | -24.2% |
| Shanghai | Women | 9.1 | 8.5 | 7.4 | 6.4 | -2.34(-2.49, - 2.19) | -29.7% |
| Shanghai | Men | 15.2 | 14.7 | 13.6 | 12.6 | -1.25(-1.37, -1.13) | -17.1% |
| Jiangsu | Both | 11.4 | 10.9 | 10.4 | 9.6 | -1.12(-1.19, -1.05) | -15.8% |
| Jiangsu | Women | 8.8 | 8.5 | 8.1 | 7.5 | -1.06(-1.18, -0.95) | -14.8% |
| Jiangsu | Men | 14.2 | 13.5 | 12.8 | 12.0 | -1.13(-1.21, -1.06) | -15.5% |
| Zhejiang | Both | 9.0 | 10.0 | 11.1 | 11.6 | 1.74(1.64, 1.84) | 28.9% |
| Zhejiang | Women | 8.0 | 8.1 | 8.2 | 8.0 | 0.02(-0.08, 0.12) | 0.0% |
| Zhejiang | Men | 10.0 | 11.9 | 13.9 | 15.4 | 2.95(2.82, 3.09) | 54.0% |
| Anhui | Both | 6.9 | 8.2 | 9.5 | 10.6 | 2.92(2.73, 3.11) | 53.6% |
| Anhui | Women | 5.2 | 6.1 | 7.1 | 7.9 | 2.81(2.68, 2.94) | 51.9% |
| Anhui | Men | 8.9 | 10.4 | 12.1 | 13.5 | 2.82(2.69, 2.95) | 51.7% |
| Fujian | Both | 4.4 | 5.6 | 6.7 | 7.5 | 3.67(3.39, 3.95) | 70.5% |
| Fujian | Women | 3.7 | 4.5 | 5.3 | 5.7 | 2.95(2.70, 3.19) | 54.1% |
| Fujian | Men | 5.2 | 6.8 | 8.3 | 9.5 | 4.19(3.93, 4.45) | 82.7% |
| Jiangxi | Both | 5.9 | 5.8 | 5.7 | 5.5 | -0.47(-0.57, -0.37) | -6.8% |
| Jiangxi | Women | 3.4 | 3.6 | 3.7 | 3.7 | 0.53(0.42, 0.65) | 8.8% |
| Jiangxi | Men | 8.8 | 8.4 | 7.9 | 7.4 | -1.15(-1.18, -1.11) | -15.9% |
| Shandong | Both | 7.3 | 6.7 | 6.2 | 5.5 | -1.87(-2.01, -1.74) | -24.7% |
| Shandong | Women | 4.3 | 4.1 | 3.9 | 3.7 | -0.98(-1.07, -0.89) | -14.0% |
| Shandong | Men | 10.7 | 9.6 | 8.6 | 7.6 | -2.27(-2.39, -2.15) | -29.0% |
| Henan | Both | 4.6 | 5.0 | 5.3 | 5.5 | 1.33(1.17, 1.49) | 19.6% |
| Henan | Women | 3.8 | 3.9 | 3.9 | 3.9 | 0.17(0.17, 0.17) | 2.6% |
| Henan | Men | 5.5 | 6.2 | 6.9 | 7.4 | 2.08(1.93, 2.24) | 34.5% |
| Hubei | Both | 4.7 | 5.2 | 5.6 | 6.0 | 1.64(1.53, 1.75) | 27.7% |
| Hubei | Women | 3.7 | 4.0 | 4.2 | 4.4 | 1.16(1.06, 1.26) | 18.9% |
| Hubei | Men | 5.8 | 6.4 | 7.1 | 7.8 | 2.03(1.93, 2.14) | 34.5% |
| Hunan | Both | 5.2 | 5.4 | 5.4 | 5.5 | 0.39(0.29, 0.50) | 5.8% |
| Hunan | Women | 2.7 | 2.8 | 2.8 | 2.8 | 0.24(0.24, 0.24) | 3.7% |
| Hunan | Men | 7.7 | 8.0 | 8.1 | 8.2 | 0.42(0.33, 0.51) | 6.5% |
| Guangdong | Both | 4.6 | 4.9 | 5.2 | 5.2 | 0.84(0.69, 0.99) | 13.0% |
| Guangdong | Women | 4.0 | 4.2 | 4.3 | 4.3 | 0.42(0.26, 0.58) | 7.5% |
| Guangdong | Men | 5.3 | 5.7 | 6.1 | 6.3 | 1.15(1.01, 1.29) | 18.9% |
| Guangxi | Both | 3.8 | 3.7 | 3.7 | 3.5 | -0.50(-0.70, -0.30) | -7.9% |
| Guangxi | Women | 2.5 | 2.5 | 2.4 | 2.2 | -0.80(-0.97, -0.63) | -12.0% |
| Guangxi | Men | 5.3 | 5.3 | 5.2 | 5.1 | -0.24(-0.33, -0.16) | -3.8% |
| Hainan | Both | 2.4 | 3.1 | 4.1 | 4.9 | 4.93(4.60, 5.26) | 104.2% |
| Hainan | Women | 2.8 | 2.8 | 3.2 | 3.6 | 1.77(1.33, 2.22) | 28.6% |
| Hainan | Men | 1.6 | 3.3 | 5.0 | 6.5 | 9.85(9.33, 10.38) | 306.3% |
| Chongqing | Both | 4.7 | 5.6 | 6.3 | 6.8 | 2.53(2.34, 2.72) | 44.7% |
| Chongqing | Women | 2.8 | 3.7 | 4.4 | 5.0 | 3.98(3.58, 4.39) | 78.6% |
| Chongqing | Men | 6.7 | 7.5 | 8.2 | 8.8 | 1.83(1.73, 1.93) | 31.3% |
| Sichuan | Both | 5.4 | 6.2 | 6.8 | 7.3 | 2.07(1.80, 2.34) | 35.2% |
| Sichuan | Women | 3.6 | 4.3 | 5.0 | 5.4 | 2.65(2.18, 3.11) | 50.0% |
| Sichuan | Men | 7.3 | 8.1 | 8.8 | 9.2 | 1.58(1.40, 1.76) | 26.0% |
| Guizhou | Both | 3.7 | 4.8 | 5.9 | 6.6 | 3.93(3.49, 4.37) | 78.4% |
| Guizhou | Women | 2.4 | 3.0 | 3.6 | 3.9 | 3.29(2.88, 3.70) | 62.5% |
| Guizhou | Men | 5.1 | 6.8 | 8.6 | 9.8 | 4.41(4.17, 4.65) | 92.2% |
| Yunnan | Both | 3.9 | 4.1 | 4.3 | 4.4 | 0.79(0.56, 1.02) | 12.8% |
| Yunnan | Women | 3.0 | 3.1 | 3.2 | 3.2 | 0.43(0.31, 0.56) | 6.7% |
| Yunnan | Men | 4.9 | 5.3 | 5.6 | 5.9 | 1.30(1.16, 1.45) | 20.4% |
| Tibet | Both | 1.4 | 2.1 | 3.6 | 4.9 | 8.87(8.41, 9.32) | 250.0% |
| Tibet | Women | 0.8 | 1.7 | 3.4 | 4.8 | 12.66(11.74, 13.59) | 500.0% |
| Tibet | Men | 2.2 | 2.6 | 3.9 | 5.0 | 5.95(5.15, 6.75) | 127.3% |
| Shaanxi | Both | 5.9 | 6.9 | 7.8 | 8.5 | 2.47(2.40, 2.55) | 44.1% |
| Shaanxi | Women | 4.4 | 5.0 | 5.5 | 6.0 | 2.12(2.03, 2.22) | 36.4% |
| Shaanxi | Men | 7.5 | 9.0 | 10.2 | 11.2 | 2.69(2.59, 2.80) | 49.3% |
| Gansu | Both | 6.9 | 7.4 | 7.8 | 7.7 | 0.79(0.55, 1.04) | 11.6% |
| Gansu | Women | 4.1 | 4.5 | 4.9 | 5.0 | 1.33(1.15, 1.52) | 22.0% |
| Gansu | Men | 9.8 | 10.3 | 10.7 | 10.8 | 0.66(0.57, 0.75) | 10.2% |
| Qinghai | Both | 9.2 | 9.2 | 9.0 | 8.8 | -0.30(-0.37, -0.23) | -4.3% |
| Qinghai | Women | 6.6 | 6.5 | 6.3 | 6.0 | -0.64(-0.75, -0.53) | -9.1% |
| Qinghai | Men | 12.0 | 12.1 | 11.9 | 11.8 | -0.16(-0.22, -0.09) | -1.7% |
| Ningxia | Both | 7.4 | 7.8 | 7.9 | 7.7 | 0.30(0.11, 0.49) | 4.1% |
| Ningxia | Women | 6.2 | 6.1 | 5.8 | 5.4 | -0.95(-1.09, -0.81) | -12.9% |
| Ningxia | Men | 8.4 | 9.4 | 10.0 | 10.4 | 1.44(1.41, 1.46) | 23.8% |
| Xinjiang | Both | 11.2 | 10.4 | 9.4 | 8.4 | -1.88(-1.97, -1.80) | -25.0% |
| Xinjiang | Women | 6.5 | 6.3 | 5.8 | 5.4 | -1.23(-1.35, -1.11) | -16.9% |
| Xinjiang | Men | 15.2 | 14.1 | 12.6 | 11.3 | -1.96(-2.06, -1.86) | -25.7% |

| **Table S2** The age-standardized YLL rates (1/100,000) by province, gender, 2005-2020 | | | | | | | |
| --- | --- | --- | --- | --- | --- | --- | --- |
| **Provinces** | **Gender** | **2005** | **2010** | **2015** | **2020** | **AAPC** | **Percent Change** |
| China | Both | 146.2 | 152.3 | 157 | 158.6 | 0.54(0.47, 0.62) | 8.5% |
| China | Women | 102.3 | 106 | 108.3 | 108.7 | 0.42(0.36, 0.47) | 6.3% |
| China | Men | 192.2 | 200.7 | 207.5 | 212.4 | 0.66(0.60, 0.72) | 10.5% |
| Beijing | Both | 149.3 | 156.9 | 163.4 | 170.7 | 0.91(0.85, 0.97) | 14.3% |
| Beijing | Women | 107.3 | 112 | 115.6 | 119.3 | 0.72(0.67, 0.78) | 11.2% |
| Beijing | Men | 199 | 208.6 | 216.8 | 228.1 | 0.92(0.86, 0.99) | 14.6% |
| Tianjin | Both | 153.3 | 169.9 | 186.2 | 205.1 | 1.97(1.86, 2.08) | 33.8% |
| Tianjin | Women | 142.9 | 149.8 | 155.4 | 158.8 | 0.70(0.66, 0.73) | 11.1% |
| Tianjin | Men | 165.9 | 193.7 | 223.3 | 251.6 | 2.82(2.78, 2.85) | 51.7% |
| Hebei | Both | 179 | 176.9 | 175.8 | 169.4 | -0.33(-0.42, -0.24) | -5.4% |
| Hebei | Women | 109.2 | 102.9 | 98.1 | 93 | -1.07(-1.16, -0.99) | -14.8% |
| Hebei | Men | 254.2 | 257.4 | 258.9 | 256.2 | 0.05(0.02, 0.08) | 0.8% |
| Shanxi | Both | 137.9 | 147.3 | 152.2 | 154.3 | 0.77(0.66, 0.88) | 11.9% |
| Shanxi | Women | 82.9 | 88.8 | 93.9 | 96.8 | 1.04(0.98, 1.11) | 16.8% |
| Shanxi | Men | 193.8 | 206.2 | 211.8 | 214.8 | 0.70(0.67, 0.74) | 10.8% |
| Inner Mongolia | Both | 163.8 | 189.2 | 212.2 | 230.9 | 2.33(2.25, 2.40) | 41.0% |
| Inner Mongolia | Women | 118.2 | 134.1 | 148.6 | 159.2 | 2.03(1.92, 2.14) | 34.7% |
| Inner Mongolia | Men | 207.9 | 245.4 | 280 | 307.7 | 2.65(2.57, 2.72) | 48.0% |
| Liaoning | Both | 203.2 | 198.8 | 194.4 | 187.5 | -0.54(-0.57, -0.50) | -7.7% |
| Liaoning | Women | 136.6 | 139.9 | 141.2 | 141 | 0.21(0.17, 0.24) | 3.2% |
| Liaoning | Men | 271.7 | 260.3 | 250.9 | 239.3 | -0.85(-0.89, -0.80) | -11.9% |
| Jilin | Both | 147.5 | 180.6 | 211.7 | 239.2 | 3.30(3.20, 3.40) | 62.2% |
| Jilin | Women | 90.8 | 112.3 | 132.4 | 151.7 | 3.46(3.39, 3.54) | 67.1% |
| Jilin | Men | 204.8 | 252 | 295.9 | 337.9 | 3.42(3.30, 3.54) | 65.0% |
| Heilongjiang | Both | 212.4 | 207.3 | 203.8 | 199 | -0.43(-0.48, -0.38) | -6.3% |
| Heilongjiang | Women | 144.8 | 144.9 | 145.6 | 144.5 | -0.02(-0.04, 0.01) | -0.2% |
| Heilongjiang | Men | 279.4 | 271.8 | 267.4 | 261.3 | -0.45(-0.48, -0.42) | -6.5% |
| Shanghai | Both | 236.8 | 217.8 | 192 | 163.5 | -2.43(-2.61, -2.25) | -31.0% |
| Shanghai | Women | 163.3 | 151.2 | 132.7 | 114.2 | -2.32(-2.42, -2.23) | -30.1% |
| Shanghai | Men | 317.1 | 291.8 | 256.4 | 222.6 | -2.29(-2.37, -2.21) | -29.8% |
| Jiangsu | Both | 257.1 | 240.4 | 222.3 | 200.1 | -1.63(--1.70, -1.56) | -22.2% |
| Jiangsu | Women | 193.8 | 182.3 | 167.8 | 150.5 | -1.67(-1.74, -1.60) | -22.3% |
| Jiangsu | Men | 324.7 | 301.6 | 279.4 | 254.1 | -1.62(-1.65, -1.59) | -21.7% |
| Zhejiang | Both | 199.3 | 218.2 | 239 | 248.6 | 1.49(1.41, 1.56) | 24.7% |
| Zhejiang | Women | 171 | 171.9 | 172 | 165.9 | -0.19(-0.24, -0.15) | -3.0% |
| Zhejiang | Men | 227.4 | 264.9 | 302.6 | 332.9 | 2.56(2.43, 2.68) | 46.4% |
| Anhui | Both | 154.9 | 184.7 | 213.2 | 238.4 | 2.93(2.78, 3.08) | 53.9% |
| Anhui | Women | 120 | 137.2 | 154.5 | 169.3 | 2.35(2.26, 2.44) | 41.1% |
| Anhui | Men | 193.7 | 232.9 | 273.9 | 311 | 3.23(3.12, 3.33) | 60.6% |
| Fujian | Both | 111.9 | 134.8 | 155.8 | 168.6 | 2.82(2.63, 3.00) | 50.7% |
| Fujian | Women | 94.2 | 107.8 | 119.9 | 123.8 | 1.84(1.73, 1.94) | 31.4% |
| Fujian | Men | 130.7 | 162.8 | 193.3 | 216.5 | 3.43(3.33, 3.53) | 65.6% |
| Jiangxi | Both | 118.9 | 118 | 116.6 | 113.1 | -0.28(-0.37, -0.20) | -4.9% |
| Jiangxi | Women | 69.4 | 71.9 | 73.5 | 74.9 | 0.52(0.48, 0.56) | 7.9% |
| Jiangxi | Men | 173.4 | 166.9 | 160.1 | 154.4 | -0.76(-0.78, -0.74) | -11.0% |
| Shandong | Both | 151.6 | 138.9 | 127 | 113.2 | -1.94(-2.01, -1.86) | -25.3% |
| Shandong | Women | 77.7 | 74.8 | 72 | 68.4 | -0.84(-0.87, -0.82) | -12.0% |
| Shandong | Men | 230.5 | 206.9 | 184.8 | 160.7 | -2.39(-2.44, -2.33) | -30.3% |
| Henan | Both | 100.7 | 107.9 | 113.5 | 115.9 | 0.96(0.87, 1.04) | 15.1% |
| Henan | Women | 79.7 | 81.1 | 81.4 | 80.3 | 0.10(0.03, 0.16) | 0.8% |
| Henan | Men | 123 | 136.3 | 148.2 | 156 | 1.60(1.55, 1.65) | 26.8% |
| Hubei | Both | 104.3 | 116.1 | 127.2 | 136.8 | 1.83(1.76, 1.89) | 31.2% |
| Hubei | Women | 81.8 | 88.5 | 94.4 | 99.4 | 1.31(1.26, 1.37) | 21.5% |
| Hubei | Men | 127.4 | 143.9 | 160.9 | 176.8 | 2.21(2.18, 2.23) | 38.8% |
| Hunan | Both | 120 | 121.6 | 121.1 | 119 | -0.02(-0.09, 0.05) | -0.8% |
| Hunan | Women | 64.3 | 64.7 | 63.9 | 62.8 | -0.17(-0.20, -0.14) | -2.3% |
| Hunan | Men | 175.2 | 177.6 | 177.8 | 176.3 | 0.05(0.02, 0.08) | 0.6% |
| Guangdong | Both | 100.3 | 106.6 | 112.3 | 113.3 | 0.82(0.74, 0.91) | 13.0% |
| Guangdong | Women | 90.9 | 93 | 93.6 | 92 | 0.09(0.01, 0.17) | 1.2% |
| Guangdong | Men | 112.1 | 122 | 130.1 | 135.9 | 1.29(1.24, 1.35) | 21.2% |
| Guangxi | Both | 90 | 87.4 | 84.4 | 78.9 | -0.85(-0.92, -0.79) | -12.3% |
| Guangxi | Women | 68.6 | 63.2 | 57.7 | 51.1 | -1.94(-1.98, -1.90) | -25.5% |
| Guangxi | Men | 115.6 | 114.6 | 113.6 | 109.7 | -0.34(-0.39, -0.29) | -5.1% |
| Hainan | Both | 54.2 | 71.8 | 95.2 | 114.3 | 5.19(4.99, 5.40) | 110.9% |
| Hainan | Women | 49.9 | 56.9 | 73.3 | 87.7 | 3.92(3.73, 4.10) | 75.8% |
| Hainan | Men | 51.7 | 83.4 | 116 | 144 | 7.14(6.92, 7.35) | 178.5% |
| Chongqing | Both | 106.1 | 122.4 | 137.1 | 146.2 | 2.22(2.04, 2.41) | 37.8% |
| Chongqing | Women | 56.8 | 74.5 | 89.9 | 102 | 4.01(3.81, 4.21) | 79.6% |
| Chongqing | Men | 154.2 | 168.5 | 181.7 | 191.8 | 1.47(1.43, 1.51) | 24.4% |
| Sichuan | Both | 118.9 | 136.1 | 149.9 | 158.9 | 1.99(1.78, 2.19) | 33.6% |
| Sichuan | Women | 76.8 | 91 | 104.2 | 111.8 | 2.54(2.44, 2.63) | 45.6% |
| Sichuan | Men | 160.8 | 180.3 | 197.2 | 207.6 | 1.72(1.60, 1.84) | 29.1% |
| Guizhou | Both | 97.3 | 113.4 | 129.4 | 136.3 | 2.28(2.13, 2.42) | 40.1% |
| Guizhou | Women | 70.4 | 76.1 | 81.2 | 80.5 | 0.88(0.80, 0.97) | 14.3% |
| Guizhou | Men | 125.6 | 152.6 | 181.5 | 199.1 | 3.09(2.96, 3.22) | 58.5% |
| Yunnan | Both | 94.4 | 99 | 102.2 | 103.6 | 0.64(0.56, 0.71) | 9.7% |
| Yunnan | Women | 70.4 | 73.1 | 75.2 | 75.5 | 0.48(0.43, 0.53) | 7.2% |
| Yunnan | Men | 118.9 | 125.8 | 131.2 | 134.1 | 0.82(0.78, 0.85) | 12.8% |
| Tibet | Both | 46.8 | 57.2 | 93.8 | 124.9 | 7.01(6.52, 7.50) | 166.9% |
| Tibet | Women | 25.9 | 44.4 | 82.7 | 114.1 | 10.56(10.07, 11.06) | 340.5% |
| Tibet | Men | 68.5 | 70.6 | 104.6 | 134 | 4.78(4.36, 5.20) | 95.6% |
| Shaanxi | Both | 137.3 | 150.9 | 162.7 | 171.5 | 1.54(1.46, 1.61) | 24.9% |
| Shaanxi | Women | 98.8 | 107.6 | 116.5 | 124.1 | 1.55(1.52, 1.58) | 25.6% |
| Shaanxi | Men | 175.6 | 195.5 | 210.7 | 223 | 1.63(1.54, 1.71) | 27.0% |
| Gansu | Both | 154 | 162.8 | 170.5 | 167.8 | 0.59(0.41, 0.78) | 9.0% |
| Gansu | Women | 95.8 | 104.2 | 111.3 | 112.2 | 1.08(0.99, 1.18) | 17.1% |
| Gansu | Men | 211.5 | 221.1 | 228.3 | 227.3 | 0.48(0.43, 0.54) | 7.5% |
| Qinghai | Both | 227.5 | 214.5 | 198.3 | 186.7 | -1.30(-1.41, -1.19) | -17.9% |
| Qinghai | Women | 161.1 | 147.4 | 132.4 | 116.9 | -2.13(-2.21, -2.06) | -27.4% |
| Qinghai | Men | 294.9 | 284.5 | 270.1 | 259.9 | -0.84(-0.89, -0.80) | -11.9% |
| Ningxia | Both | 191.8 | 188 | 180.6 | 168 | -0.87(-0.95, -0.80) | -12.4% |
| Ningxia | Women | 160 | 147.7 | 131.5 | 115.4 | -2.20(-2.33, -2.07) | -27.9% |
| Ningxia | Men | 222.6 | 228.8 | 230 | 224.6 | 0.05(0.00, 0.11) | 0.9% |
| Xinjiang | Both | 229.3 | 208.1 | 184.9 | 162.2 | -2.24(-2.33, -2.14) | -29.3% |
| Xinjiang | Women | 130.2 | 123 | 113.4 | 104.2 | -1.50(-1.53, -1.46) | -20.0% |
| Xinjiang | Men | 316.7 | 286.5 | 252.5 | 221 | -2.36(-2.42, -2.30) | -30.2% |

| **Table S3** The age-standardized mortality rates (1/100,000) by province, urban-rural, 2005-2020 | | | | | | | |
| --- | --- | --- | --- | --- | --- | --- | --- |
| **Provinces** | **Urban-Rural** | **2005** | **2010** | **2015** | **2020** | **AAPC** | **Percent Change** |
| China | Both | 6.6 | 7.0 | 7.3 | 7.4 | 0.76(0.58, 0.95) | 12.1% |
| China | Urban | 9.8 | 10.4 | 8.8 | 8.4 | -1.11(-2.12, -0.10) | -14.3% |
| China | Rural | 4.7 | 5.0 | 6.3 | 6.7 | 2.59(1.54, 3.64) | 42.6% |
| Beijing | Both | 7.7 | 7.9 | 8.0 | 8.1 | 0.37(0.29, 0.46) | 5.2% |
| Beijing | Urban | 7.9 | 8.2 | 8.1 | 8.2 | 0.26(-0.21, 0.73) | 3.8% |
| Beijing | Rural | 5.8 | 5.9 | 7.7 | 7.5 | 1.54(-2.33, 5.56) | 29.3% |
| Tianjin | Both | 6.6 | 7.5 | 8.4 | 9.4 | 2.39(2.26, 2.53) | 42.4% |
| Tianjin | Urban | 11.2 | 10.9 | 9.0 | 10.1 | -0.67(-3.84, 2.60) | -9.8% |
| Tianjin | Rural | 3.6 | 5.4 | 5.0 | 5.0 | 4.03(1.83, 6.29) | 38.9% |
| Hebei | Both | 7.7 | 8.0 | 8.2 | 8.1 | 0.35(0.25, 0.44) | 5.2% |
| Hebei | Urban | 11.9 | 10.8 | 11.9 | 10.3 | -0.36(-1.70, 1.01) | -13.4% |
| Hebei | Rural | 5.7 | 6.6 | 7.4 | 7.6 | 2.27(1.61, 2.94) | 33.3% |
| Shanxi | Both | 6.5 | 7.0 | 7.2 | 7.3 | 0.78(0.63, 0.93) | 12.3% |
| Shanxi | Urban | 8.8 | 14.2 | 8.3 | 8.3 | -0.23(-5.02, 4.80) | -5.7% |
| Shanxi | Rural | 5.1 | 2.3 | 6.7 | 6.8 | 1.03(-7.53, 10.38) | 33.3% |
| Inner Mongolia | Both | 7.4 | 8.8 | 10.0 | 11.0 | 2.70(2.53, 2.87) | 48.6% |
| Inner Mongolia | Urban | 10.0 | 10.5 | 10.3 | 10.6 | -0.25(-0.99, 0.49) | 6.0% |
| Inner Mongolia | Rural | 4.2 | 6.6 | 9.7 | 11.2 | 7.01(4.99, 9.06) | 166.7% |
| Liaoning | Both | 9.6 | 9.4 | 9.2 | 8.8 | -0.57(-0.66, -0.49) | -8.3% |
| Liaoning | Urban | 13.3 | 13.7 | 11.0 | 10.4 | -1.96(-.64, -1.27) | -21.8% |
| Liaoning | Rural | 7.0 | 6.4 | 8.2 | 8.0 | 1.39(0.40, 2.39) | 14.3% |
| Jilin | Both | 7.2 | 8.9 | 10.5 | 11.9 | 3.54(3.29, 3.62) | 65.3% |
| Jilin | Urban | 6.7 | 8.9 | 9.2 | 11.7 | 3.05(1.54, 4.59) | 74.6% |
| Jilin | Rural | 7.4 | 8.9 | 10.9 | 12.0 | 3.78(2.89, 4.67) | 62.2% |
| Heilongjiang | Both | 9.3 | 9.1 | 9.1 | 8.9 | -0.33(-0.55, -0.11) | -4.3% |
| Heilongjiang | Urban | 10.4 | 10.6 | 9.6 | 9.0 | -1.00(-1.59, -0.41) | -13.5% |
| Heilongjiang | Rural | 7.9 | 7.2 | 8.6 | 8.7 | 1.27(0.50, 2.03) | 10.1% |
| Shanghai | Both | 12.0 | 11.4 | 10.3 | 9.1 | -1.83(-1.95, -1.71) | -24.2% |
| Shanghai | Urban | 12.0 | 11.4 | 10.3 | 9.1 | -1.83(-1.95, -1.71) | -24.2% |
| Shanghai | Rural | 0.0 | 0.0 | 0.0 | 0.0 | 0 | — |
| Jiangsu | Both | 11.4 | 10.9 | 10.4 | 9.6 | -1.12(-1.19, -1.05) | -15.8% |
| Jiangsu | Urban | 12.3 | 12.9 | 8.7 | 9.4 | -1.37(-3.39, 0.68) | -23.6% |
| Jiangsu | Rural | 10.8 | 9.7 | 11.4 | 9.8 | -0.87(-2.12, 0.40) | -9.3% |
| Zhejiang | Both | 9.0 | 10.0 | 11.1 | 11.6 | 1.74(1.64, 1.84) | 28.9% |
| Zhejiang | Urban | 11.2 | 11.6 | 12.5 | 13.8 | 0.83(-0.51, 2.18) | 23.2% |
| Zhejiang | Rural | 7.9 | 9.2 | 10.9 | 11.3 | 2.63(1.92, 3.34) | 43.0% |
| Anhui | Both | 6.9 | 8.2 | 9.5 | 10.6 | 2.92(2.73, 3.11) | 53.6% |
| Anhui | Urban | 8.0 | 12.0 | 9.9 | 9.7 | 1.49(-1.53, 4.59) | 21.3% |
| Anhui | Rural | 6.2 | 6.1 | 9.2 | 11.1 | 3.49(0.66, 6.40) | 79.0% |
| Fujian | Both | 4.4 | 5.6 | 6.7 | 7.5 | 3.67(3.39, 3.95) | 70.5% |
| Fujian | Urban | 6.6 | 6.4 | 7.8 | 8.1 | 0.28(-4.75, 5.57) | 22.7% |
| Fujian | Rural | 3.7 | 5.4 | 6.0 | 7.0 | 4.80(2.68, 6.97) | 89.2% |
| Jiangxi | Both | 5.9 | 5.8 | 5.7 | 5.5 | -0.47(-0.57, -0.37) | -6.8% |
| Jiangxi | Urban | 3.9 | 8.2 | 6.5 | 6.8 | 3.85(-0.04, 7.89) | 74.4% |
| Jiangxi | Rural | 8.0 | 3.4 | 5.3 | 4.8 | -3.97(-8.74, 1.05) | -40.0% |
| Shandong | Both | 7.3 | 6.7 | 6.2 | 5.5 | -1.87(-2.01, -1.74) | -24.7% |
| Shandong | Urban | 8.9 | 8.0 | 6.6 | 5.6 | -3.02(-3.35, -2.69) | -37.1% |
| Shandong | Rural | 5.6 | 5.3 | 5.8 | 5.5 | 0.07(-0.37, 0.52) | -1.8% |
| Henan | Both | 4.6 | 5.0 | 5.3 | 5.5 | 1.33(1.17, 1.49) | 19.6% |
| Henan | Urban | 4.7 | 5.9 | 7.2 | 6.7 | 2.07(0.37, 3.81) | 42.6% |
| Henan | Rural | 4.5 | 4.7 | 4.7 | 5.0 | 0.88(0.25, 1.51) | 11.1% |
| Hubei | Both | 4.7 | 5.2 | 5.6 | 6.0 | 1.64(1.53, 1.75) | 27.7% |
| Hubei | Urban | 10.7 | 10.7 | 8.0 | 6.8 | -2.96(-3.82, -2.09) | -36.4% |
| Hubei | Rural | 2.2 | 2.9 | 4.8 | 5.7 | 6.52(5.40, 7.66) | 159.1% |
| Hunan | Both | 5.2 | 5.4 | 5.4 | 5.5 | 0.39(0.29, 0.50) | 5.8% |
| Hunan | Urban | 7.1 | 7.0 | 8.3 | 7.2 | 0.75(-0.47, 1.98) | 1.4% |
| Hunan | Rural | 4.4 | 4.7 | 4.4 | 4.7 | 0.31(-0.40, 1.03) | 6.8% |
| Guangdong | Both | 4.6 | 4.9 | 5.2 | 5.2 | 0.84(0.69, 0.99) | 13.0% |
| Guangdong | Urban | 8.9 | 9.5 | 6.1 | 5.7 | -2.64(-4.97, -0.26) | -36.0% |
| Guangdong | Rural | 1.8 | 1.9 | 3.9 | 4.6 | 9.06(6.30, 11.88) | 155.6% |
| Guangxi | Both | 3.8 | 3.7 | 3.7 | 3.5 | -0.50(-0.70, -0.30) | -7.9% |
| Guangxi | Urban | 8.9 | 5.5 | 5.6 | 5.3 | -4.39(-6.29, -2.45) | -40.4% |
| Guangxi | Rural | 2.4 | 3.3 | 2.9 | 2.7 | 1.90(-0.97, 4.85) | 12.5% |
| Hainan | Both | 2.4 | 3.1 | 4.1 | 4.9 | 4.93(4.60, 5.26) | 104.2% |
| Hainan | Urban | 1.4 | 2.8 | 3.7 | 7.3 | 7.31(3.13, 11.65) | 421.4% |
| Hainan | Rural | 3.7 | 3.6 | 4.3 | 3.7 | 1.64(-1.48, 4.86) | 0.0% |
| Chongqing | Both | 4.7 | 5.6 | 6.3 | 6.8 | 2.53(2.34, 2.72) | 44.7% |
| Chongqing | Urban | 5.0 | 6.2 | 7.1 | 7.2 | 2.43(1.59, 3.28) | 44.0% |
| Chongqing | Rural | 4.2 | 4.5 | 3.5 | 5.0 | 0.34(-3.09, 3.88) | 19.0% |
| Sichuan | Both | 5.4 | 6.2 | 6.8 | 7.3 | 2.07(1.80, 2.34) | 35.2% |
| Sichuan | Urban | 11.3 | 10.9 | 7.3 | 6.8 | -4.00(-5.31, -2.66) | -39.8% |
| Sichuan | Rural | 4.1 | 5.1 | 6.6 | 7.4 | 4.36(3.67, 5.06) | 80.5% |
| Guizhou | Both | 3.7 | 4.8 | 5.9 | 6.6 | 3.93(3.49, 4.37) | 78.4% |
| Guizhou | Urban | 9.0 | 9.5 | 8.4 | 8.0 | 0.98(-1.21, 3.23) | -11.1% |
| Guizhou | Rural | 1.6 | 2.9 | 5.2 | 5.7 | 9.70(5.00, 14.61) | 256.3% |
| Yunnan | Both | 3.9 | 4.1 | 4.3 | 4.4 | 0.79(0.56, 1.02) | 12.8% |
| Yunnan | Urban | 13.3 | 8.7 | 4.9 | 4.6 | -6.91(-9.73, -4.00) | -65.4% |
| Yunnan | Rural | 1.9 | 3.2 | 4.2 | 4.4 | 5.71(3.89, 7.56) | 131.6% |
| Tibet | Both | 1.4 | 2.1 | 3.6 | 4.9 | 8.87(8.41, 9.32) | 250.0% |
| Tibet | Urban | 1.5 | 2.5 | 5.7 | 7.8 | 12.56(9.59, 15.61) | 420.0% |
| Tibet | Rural | 1.3 | 1.5 | 2.3 | 3.1 | 4.53(2.65, 6.43) | 138.5% |
| Shaanxi | Both | 5.9 | 6.9 | 7.8 | 8.5 | 2.47(2.40, 2.55) | 44.1% |
| Shaanxi | Urban | 16.7 | 13.7 | 8.5 | 9.0 | -3.77(-6.67, -0.78) | -46.1% |
| Shaanxi | Rural | 3.8 | 5.6 | 6.7 | 7.6 | 5.39(1.95, 8.94) | 100.0% |
| Gansu | Both | 6.9 | 7.4 | 7.8 | 7.7 | 0.79(0.55, 1.04) | 11.6% |
| Gansu | Urban | 6.7 | 4.3 | 10.3 | 8.6 | 3.23(-0.15, 6.73) | 28.4% |
| Gansu | Rural | 7.0 | 9.0 | 4.0 | 6.4 | -0.49(-8.00, 7.63) | -8.6% |
| Qinghai | Both | 9.2 | 9.2 | 9.0 | 8.8 | -0.30(-0.37, -0.23) | -4.3% |
| Qinghai | Urban | 13.6 | 13.9 | 13.3 | 13.3 | 0.72(-1.27, 2.75) | -2.2% |
| Qinghai | Rural | 6.6 | 6.4 | 6.5 | 6.2 | -1.20(-2.92, 0.56) | -6.1% |
| Ningxia | Both | 7.4 | 7.8 | 7.9 | 7.7 | 0.30(0.11, 0.49) | 4.1% |
| Ningxia | Urban | 11.0 | 12.9 | 9.0 | 7.9 | -3.54(-5.10, -1.95) | -28.2% |
| Ningxia | Rural | 4.4 | 3.8 | 6.7 | 7.5 | 6.52(3.69, 9.43) | 70.5% |
| Xinjiang | Both | 11.2 | 10.4 | 9.4 | 8.4 | -1.88(-1.97, -1.80) | -25.0% |
| Xinjiang | Urban | 35.2 | 30.3 | 19.4 | 15.3 | -5.79(-6.76, -4.81) | -56.5% |
| Xinjiang | Rural | 2.5 | 3.4 | 6.6 | 6.5 | 7.30(3.22, 11.54) | 160.0% |

| **Table S4** The age-standardized YLL rate (1/100,000) by province, urban-rural, 2005-2020 | | | | | | | |
| --- | --- | --- | --- | --- | --- | --- | --- |
| **Provinces** | **Urban-Rural** | **2005** | **2010** | **2015** | **2020** | **AAPC** | **Percent Change** |
| China | Both | 146.2 | 152.3 | 157.0 | 158.6 | 0.54(0.47, 0.62) | 8.5% |
| China | Urban | 218.1 | 225.9 | 188.5 | 178.9 | -1.39(-2.37, -0.39) | -18.0% |
| China | Rural | 103.2 | 108.2 | 136.1 | 144.0 | 2.39(1.37, 3.42) | 39.5% |
| Beijing | Both | 149.3 | 156.9 | 163.4 | 170.7 | 0.91(0.85, 0.97) | 14.3% |
| Beijing | Urban | 153.9 | 161.7 | 164.1 | 172.3 | 0.79(0.34, 1.24) | 12.0% |
| Beijing | Rural | 111.6 | 117.3 | 157.3 | 157.9 | 2.11(-1.80, 6.19) | 41.5% |
| Tianjin | Both | 153.3 | 169.9 | 186.2 | 205.1 | 1.97(1.86, 2.08) | 33.8% |
| Tianjin | Urban | 259.0 | 245.5 | 198.8 | 220.2 | -1.03(-4.29, 2.34) | -15.0% |
| Tianjin | Rural | 84.4 | 121.5 | 111.6 | 109.1 | 3.65(1.47, 5.88) | 29.3% |
| Hebei | Both | 179.0 | 176.9 | 175.8 | 169.4 | -0.33(-0.42, -0.24) | -5.4% |
| Hebei | Urban | 276.2 | 239.1 | 255.6 | 215.2 | -1.02(-2.34, 0.32) | -22.1% |
| Hebei | Rural | 132.4 | 147.6 | 157.9 | 159.4 | 1.55(0.89, 2.22) | 20.4% |
| Shanxi | Both | 137.9 | 147.3 | 152.2 | 154.3 | 0.77(0.66, 0.88) | 11.9% |
| Shanxi | Urban | 185.6 | 301.7 | 176.5 | 176.8 | -0.16(-4.85, 4.75) | -4.7% |
| Shanxi | Rural | 107.3 | 47.8 | 141.2 | 143.9 | 1.07(-7.44, 10.37) | 34.1% |
| Inner Mongolia | Both | 163.8 | 189.2 | 212.2 | 230.9 | 2.33(2.25, 2.40) | 41.0% |
| Inner Mongolia | Urban | 220.4 | 226.2 | 219.0 | 223.4 | -0.54(-1.29, 0.22) | 1.4% |
| Inner Mongolia | Rural | 92.5 | 142.5 | 207.2 | 236.3 | 6.67(4.69, 8.68) | 155.5% |
| Liaoning | Both | 203.2 | 198.8 | 194.4 | 187.5 | -0.54(-0.57, -0.50) | -7.7% |
| Liaoning | Urban | 281.0 | 289.8 | 233.8 | 222.2 | -1.89(-2.56, -1.21) | -20.9% |
| Liaoning | Rural | 148.7 | 134.9 | 175.1 | 170.7 | 1.46(0.48, 2.45) | 14.8% |
| Jilin | Both | 147.5 | 180.6 | 211.7 | 239.2 | 3.30(3.20, 3.40) | 62.2% |
| Jilin | Urban | 138.4 | 180.5 | 185.9 | 235.0 | 2.87(1.40, 4.37) | 69.8% |
| Jilin | Rural | 153.1 | 180.7 | 219.1 | 240.6 | 3.61(2.72, 4.51) | 57.2% |
| Heilongjiang | Both | 212.4 | 207.3 | 203.8 | 199.0 | -0.43(-0.48, -0.38) | -6.3% |
| Heilongjiang | Urban | 236.4 | 239.9 | 214.8 | 202.8 | 1.12(-1.71, -0.54) | -14.2% |
| Heilongjiang | Rural | 178.9 | 162.5 | 194.1 | 195.6 | 1.15(0.38, 1.94) | 9.3% |
| Shanghai | Both | 236.8 | 217.8 | 192.0 | 163.5 | -2.43(-2.61, -2.25) | -31.0% |
| Shanghai | Urban | 236.8 | 217.8 | 192.0 | 163.5 | -2.43(-2.61, -2.25） | -31.0% |
| Shanghai | Rural | 0.0 | 0.0 | 0.0 | 0.0 | 0 | #DIV/0! |
| Jiangsu | Both | 257.1 | 240.4 | 222.3 | 200.1 | -1.63(-1.70, -1.56) | -22.2% |
| Jiangsu | Urban | 278.2 | 284.7 | 185.8 | 194.9 | -1.93(-4.08, 0.27) | -29.9% |
| Jiangsu | Rural | 244.3 | 213.6 | 243.9 | 203.9 | -1.41(2.57, -0.24) | -16.5% |
| Zhejiang | Both | 199.3 | 218.2 | 239.0 | 248.6 | 1.49(1.41, 1.56) | 24.7% |
| Zhejiang | Urban | 248.8 | 253.7 | 269.0 | 295.4 | 0.59(-0.74, 1.94) | 18.7% |
| Zhejiang | Rural | 176.3 | 201.4 | 234.6 | 241.7 | 2.37(1.66, 3.07) | 37.1% |
| Anhui | Both | 154.9 | 184.7 | 213.2 | 238.4 | 2.93(2.78, 3.08) | 53.9% |
| Anhui | Urban | 179.5 | 270.4 | 223.1 | 218.7 | 1.50(-1.49, 4.58) | 21.8% |
| Anhui | Rural | 140.6 | 136.7 | 205.9 | 250.2 | 3.43(0.58, 6.37) | 78.0% |
| Fujian | Both | 111.9 | 134.8 | 155.8 | 168.6 | 2.82(2.63, 3.00) | 50.7% |
| Fujian | Urban | 169.3 | 153.2 | 180.8 | 182.5 | -0.56(-5.43, 4.56) | 7.8% |
| Fujian | Rural | 94.2 | 129.1 | 138.8 | 158.6 | 3.92(1.64, 3.39) | 68.4% |
| Jiangxi | Both | 118.9 | 118.0 | 116.6 | 113.1 | -0.28(-0.37, -0.20) | -4.9% |
| Jiangxi | Urban | 78.5 | 166.3 | 131.3 | 141.0 | 4.07(0.11, 8.18) | 79.6% |
| Jiangxi | Rural | 160.2 | 69.0 | 108.6 | 99.2 | -3.33(-8.62, 2.27) | -38.1% |
| Shandong | Both | 151.6 | 138.9 | 127.0 | 113.2 | -1.94(-2.01, -1.86) | -25.3% |
| Shandong | Urban | 184.2 | 165.7 | 135.9 | 114.1 | -3.10(-3.44, -2.75) | -38.1% |
| Shandong | Rural | 117.1 | 110.5 | 120.1 | 112.4 | -0.02(-0.45, 0.42) | -4.0% |
| Henan | Both | 100.7 | 107.9 | 113.5 | 115.9 | 0.96(0.87, 1.04) | 15.1% |
| Henan | Urban | 103.1 | 127.8 | 154.2 | 141.6 | 1.79(0.13, 3.47) | 37.3% |
| Henan | Rural | 100.0 | 102.3 | 99.5 | 106.2 | 0.55(-0.07, 1.18) | 6.2% |
| Hubei | Both | 104.3 | 116.1 | 127.2 | 136.8 | 1.83(1.76, 1.89) | 31.2% |
| Hubei | Urban | 238.3 | 240.3 | 181.0 | 154.5 | -2.81(-3.68, -1.93) | -35.2% |
| Hubei | Rural | 49.6 | 65.6 | 108.4 | 130.0 | 6.65(5.49, 7.82) | 162.1% |
| Hunan | Both | 120.0 | 121.6 | 121.1 | 119.0 | -0.02(-0.09, 0.05) | -0.8% |
| Hunan | Urban | 164.7 | 159.1 | 184.6 | 158.0 | 0.34(-0.88, 1.57) | -4.1% |
| Hunan | Rural | 101.6 | 106.2 | 98.8 | 102.2 | -0.09(-0.81, 0.63) | 0.6% |
| Guangdong | Both | 100.3 | 106.6 | 112.3 | 113.3 | 0.82(0.74, 0.91) | 13.0% |
| Guangdong | Urban | 195.5 | 206.5 | 132.2 | 123.0 | -2.76(-5.03, -0.42) | -37.1% |
| Guangdong | Rural | 38.9 | 42.0 | 84.8 | 99.0 | 8.96(6.24, 11.74) | 154.5% |
| Guangxi | Both | 90.0 | 87.4 | 84.4 | 78.9 | -0.85(-0.92, -0.79) | -12.3% |
| Guangxi | Urban | 213.5 | 128.6 | 127.1 | 117.3 | -4.85(-6.75, -2.91) | -45.1% |
| Guangxi | Rural | 57.0 | 76.7 | 65.1 | 60.4 | 1.53(-1.35,4.49) | 6.0% |
| Hainan | Both | 54.2 | 71.8 | 95.2 | 114.3 | 5.19(4.99, 5.40) | 110.9% |
| Hainan | Urban | 32.2 | 64.2 | 85.4 | 169.9 | 7.48(3.35, 11.77) | 427.6% |
| Hainan | Rural | 82.5 | 81.5 | 100.0 | 86.1 | 1.92(-1.18, 5.12) | 4.4% |
| Chongqing | Both | 106.1 | 122.4 | 137.1 | 146.2 | 2.22(2.04, 2.41) | 37.8% |
| Chongqing | Urban | 112.4 | 136.1 | 153.0 | 154.8 | 2.11(1.27, 2.96) | 37.7% |
| Chongqing | Rural | 94.7 | 98.2 | 76.3 | 107.5 | 0.04(-3.34, 3.55) | 13.5% |
| Sichuan | Both | 118.9 | 136.1 | 149.9 | 158.9 | 1.99(1.78, 2.19) | 33.6% |
| Sichuan | Urban | 247.6 | 239.7 | 161.1 | 149.8 | -3.97(-5.28, -2.64) | -39.5% |
| Sichuan | Rural | 89.4 | 112.5 | 144.9 | 163.0 | 4.34(3.50, 5.18) | 82.3% |
| Guizhou | Both | 97.3 | 113.4 | 129.4 | 136.3 | 2.28(2.13, 2.42) | 40.1% |
| Guizhou | Urban | 236.0 | 223.7 | 184.0 | 166.3 | -0.57(-2.71, 1.63) | -29.5% |
| Guizhou | Rural | 41.5 | 69.1 | 113.0 | 118.2 | 7.99(3.54, 12.63) | 184.8% |
| Yunnan | Both | 94.4 | 99.0 | 102.2 | 103.6 | 0.64(0.56, 0.71) | 9.7% |
| Yunnan | Urban | 320.4 | 207.7 | 115.4 | 107.0 | -7.15(-9.97, -4.24) | -66.6% |
| Yunnan | Rural | 45.0 | 75.3 | 99.2 | 102.8 | 5.54(3.64, 7.48) | 128.4% |
| Tibet | Both | 46.8 | 57.2 | 93.8 | 124.9 | 7.01(6.52, 7.50) | 166.9% |
| Tibet | Urban | 50.8 | 69.5 | 148.0 | 200.7 | 10.72(7.87, 13.65) | 295.1% |
| Tibet | Rural | 41.9 | 42.3 | 60.6 | 79.7 | 2.94(1.19, 4.72) | 90.2% |
| Shaanxi | Both | 137.3 | 150.9 | 162.7 | 171.5 | 1.54(1.46, 1.61) | 24.9% |
| Shaanxi | Urban | 384.8 | 298.1 | 177.4 | 182.0 | -4.58(-7.50, -1.58) | -52.7% |
| Shaanxi | Rural | 88.9 | 122.6 | 140.5 | 153.6 | 4.33(1.01, 7.76) | 72.8% |
| Gansu | Both | 154.0 | 162.8 | 170.5 | 167.8 | 0.59(0.41, 0.78) | 9.0% |
| Gansu | Urban | 149.7 | 94.7 | 224.9 | 186.9 | 3.08(-0.32, 6.59) | 24.8% |
| Gansu | Rural | 156.3 | 198.5 | 86.8 | 138.3 | -0.66(-8.35, 7.68) | -11.5% |
| Qinghai | Both | 227.5 | 214.5 | 198.3 | 186.7 | -1.30(-1.41, -1.19) | -17.9% |
| Qinghai | Urban | 334.8 | 325.2 | 294.6 | 283.1 | -0.29(-2.25, 1.71) | -15.4% |
| Qinghai | Rural | 163.5 | 149.2 | 143.2 | 131.1 | -2.21(-3.91, -0.47) | -19.8% |
| Ningxia | Both | 191.8 | 188.0 | 180.6 | 168.0 | -0.87(-0.95, -0.80) | -12.4% |
| Ningxia | Urban | 287.2 | 312.7 | 204.4 | 171.5 | -4.72(-6.27, -3.15) | -40.3% |
| Ningxia | Rural | 115.2 | 91.1 | 152.6 | 163.7 | 5.24(2.45, 8.10) | 42.1% |
| Xinjiang | Both | 229.3 | 208.1 | 184.9 | 162.2 | -2.24(-2.33, -2.14) | -29.3% |
| Xinjiang | Urban | 721.3 | 607.8 | 383.6 | 295.9 | -6.13(-7.09, -5.16) | -59.0% |
| Xinjiang | Rural | 52.0 | 68.5 | 129.8 | 125.6 | 6.85(2.81, 11.05) | 141.5% |
